# Porous Composite Granules with Potential Function of Bone Substitute and Simvastatin Releasing System: A Preliminary Study

**DOI:** 10.3390/ma14175068

**Published:** 2021-09-04

**Authors:** Aleksandra Laskus-Zakrzewska, Paulina Kazimierczak, Joanna Kolmas

**Affiliations:** 1 Department of Analytical Chemistry, Faculty of Pharmacy, Medical University of Warsaw, ul. Banacha 1, 02-097 Warsaw, Poland; alaskus@wum.edu.pl; 2 Independent Unit of Tissue Engineering and Regenerative Medicine, Medical University of Lublin, Chodzki 1 Street, 20-093 Lublin, Poland; paulinakazimierczak@umlub.pl

**Keywords:** simvastatin, calcium phosphates, biomaterials, zinc, selenium, drug release, hydroxyapatite, brushite, alginate, composite

## Abstract

In this work, 3D porous granules based on Zn and Se-containing calcium phosphates (CaPs) were fabricated using a droplet-extrusion technique. The composite beads varied in composition and contained two different natural polymers: sodium alginate (SA) and gelatin (GEL). To analyse and compare their physicochemical properties, such as porosity and morphology, different techniques were applied, including scanning electron microscopy (SEM), sorption of N_2_ and mercury porosimetry. Prior to the fabrication of the granules, the properties of CaPs materials, (the bioceramic base of the beads), selenium (IV)-substituted hydroxyapatite (Se-HA) and zinc-substituted dicalcium phosphate dihydrate (Zn-DCPD), were also investigated. The results of cell viability assessment showed that Se-HA powder was non-toxic to human osteoblasts (hFOB 1.19) and simultaneously exhibited high toxicity to tumour cells (Saos-2). Once the cytotoxicity assay was completed, Se-HA and Zn-DCPD were used to prepare 3D materials. The prepared porous granules were used as matrices to deliver simvastatin to bones. Simvastatin was applied in either the lipophilic form or hydrophilic form. The release kinetics of simvastatin from granules of different composition was then assessed and compared.

## 1. Introduction

Calcium phosphates (CaPs) are inorganic materials, which possess excellent bone regeneration capacity [1,2]. Their outstanding biocompatibility, osteoconductivity and osteogenicity make them one of the most commonly used biomaterials in bone tissue engineering [3]. Notable amongst these are hydroxyapatite (HA), with the general formula Ca_10_(PO_4_)_6_(OH)_2_, and dicalcium phosphate dihydrate (DCPD)-CaHPO_4_·2H_2_O, also known as brushite. Synthetic HA is a material resembling biological apatite, an inorganic compound of osseous tissue [4]. Despite its great biocompatibility, HA performs limited bioactivity and bioresorption [5]. In turn, DCPD is believed to easily convert to HA in vivo and to be an intermediate phase during hard tissue formation processes [4]. The above features of these two CaPs enable them to be used as bone substitutes, coating materials, and drug delivery matrices [6,7]. In order to meet all current requirements of orthopaedic surgery and traumatology, scientists focus not only on filling bone loss with CaPs-based biomaterials, but also on providing the employed ceramic scaffolds with additional functionalities [1,2]. Such processes not only augment the osteogenic and healing potential of these materials, they also contribute to the optimization of the mechanical strength of the artificial graft. There are several ways to functionalise CaPs-based biomaterials that have the potential to replace hard tissue. The most popular procedures include the introduction of foreign, bioactive ions into the crystal structure of CaPs, combining CaPs with natural polymers and adding a drug substance to a scaffold. When it comes to ionic substitution within the lattice, the ions employed usually perform osteogenic (Mn^2+^, Mg^2+^, Zn^2+^, SiO_4_^4−^), antibacterial (Ag^+^, Ga^3+^, Zn^2+^, Cu^2+^) or anticancer (SeO_4_^2−^, SeO_3_^2−^) activity [8,9]. Alongside replacing structural ions of CaPs, they endow the material with additional potential, which may be extremely important.

In addition to being binding materials, natural polymers (alginates, chitosan, collagen, gelatin, cellulose, etc.) improve the mechanical strength of the CaPs scaffolds and contribute to the augmentation of their bioactivity, bioresorption and bioresemblance [10,11,12].

Another way to functionalise synthetic, ceramic bone grafts is to combine them with drug substances. CaPs-based matrices have become a widely used medium for the delivery of drugs that target bones. Notable amongst them are antibiotics, anticancer agents, and antiresorptive drugs [6,13]. Apart from providing the scaffold and additional healing potential, such a solution minimises the risk of the side effects of administering a drug orally or intravenously.

Recently, a well-known hypolipemic agent, simvastatin was found to significantly affect the metabolism of hard tissue. It was discovered that, whereas the lipophilic form of simvastatin (lactone) is metabolised in the liver, its hydrophilic form (hydroxy acid) is involved in the bone healing process [14,15,16,17]. Simvastatin was proved to significantly augment bone growth by upregulating the expression of osteogenic cytokines (BMP-2, Runx2, ALP) [17,18], which activate osteoblasts. What is more, it was also observed that simvastatin protects osteoblasts from apoptosis and inhibits activity and differentiation of osteoclasts [15,17]. Another beneficial effect of simvastatin is stimulation of the neovascularization of new-forming tissue by affecting the secretion of vascular endothelial growth factor (VEGF) [18]. Several in vivo studies, together with clinical trials, confirmed the beneficial effects of simvastatin in the treatment of osteoporosis and fracture injuries [17]. Interestingly, recent studies also point to the anti-cancer properties of simvastatin [19,20,21]. It was shown in [19] that simvastatin effectively inhibited cell viability by suppressing proliferation and by inducing apoptosis in giant cell tumour of bone. In turn, Mandal et al. [22] showed promising results for use simvastatin to inhibit osteolysis in bone metastasis of breast cancer.

In this work, different CaPs-based composites were obtained, containing two components, HA and DCPD, both of which were substituted with foreign ions, SeO_3_^2−^ and Zn^2+^, respectively. Zinc and selenium (IV) were chosen as dopants due to their osteogenic relevance and anticancer activity [8,23,24,25,26,27,28,29]. The ceramic phase was then combined with natural polymers: sodium alginate (SA) and gelatin (GEL) in different amounts. The cross-linking with Zn^2+^ and glutaraldehyde (GAH) led to the fabrication of 3D materials in the form of porous granules. The different materials were used as potential matrices for the targeted delivery of simvastatin to bones. Simvastatin was applied in either its lipophilic (SIM) or hydrophilic (SVA) form. The physicochemical analysis of the obtained composites included Fourier-transform infrared spectroscopy (FT-IR), inductively-coupled plasma optical emission spectroscopy (ICP-OES), scanning electron microscopy (SEM), porosimetry, release kinetics tests, and cytotoxicity assays.

## 2. Materials and Methods

### 2.1. Preparation of CaPs Powders

Both hydroxyapatite enriched with SeO_3_^2−^ ions and dicalcium phosphate dihydrate substituted with the Zn^2+^, were synthesised using the same standard wet precipitation method [26,27,28,29]. In brief, calcium nitrate tetrahydrate (Ca(NO_3_)_2_·4H_2_O) and ammonium phosphate dibasic ((NH_4_)_2_HPO_4_) were used as a source of calcium and phosphorus, respectively. In turn, sodium selenite (Na_2_SeO_3_) and zinc nitrate hexahydrate (Zn(NO_3_)_2_·6H_2_O) were applied as the sources of foreign dopants. All the reagents were of analytical grade and purchased from Sigma Aldrich Chemicals, St. Louis, MO, USA. The reagents were weighed out so that the molar ratio (Ca + Zn)/P and Ca/(P + Se) was approximately 1.67 for HA and 1.0 for DCPD. 

In order to obtain zinc-containing DCPD (Zn-DCPD) powder, Zn/Ca molar ratio was set at 0.33. An aqueous solution of (NH_4_)_2_HPO_4_ was added drop by drop into the aqueous solution of calcium and zinc sources. The ammonia solution (a 25% solution diluted in 1:1 proportion) and nitric acid (a 63% solution diluted in 1:2 proportion) were then added in such a way that pH = 6. Once the instillation was complete, the precipitate was aged for 12 h at room temperature and then filtered and washed with distilled water. Next, it was dried at a temperature of 60 °C for 12 h and homogenised in a mortar. The obtained sample was named Zn-DCPD. 

To obtain apatite precipitate, the appropriated amounts of reagents were calculated assuming that selenite ions would substitute for orthophosphates according to the mechanism: PO_4_^3−^ + OH^−^ + Ca^2+^ ↔ SeO_3_^2−^, described elsewhere [28]. The nominal composition was thus Ca_10−x_(PO_4_)_6−x_(SeO_3_)_x_(OH)_2−x_, where x = 0.02. Then the reagents were dissolved in distilled water and the sources of phosphorus and selenium were mixed together and subsequently dropped into the calcium solution. The processes were conducted with constant stirring at room temperature. The pH was adjusted to about 10 using ammonia solution (Chempur, Poland). After the reactants were mixed, the obtained precipitate was aged during 24 h and then filtered and washed several times with distilled water. The precipitate was then dried at 90 °C for 12 h and homogenised in a mortar. The obtained sample was named Se-HA.

### 2.2. Characterization of the Powders

The identity of the powders was determined using powder X-Ray diffractometry (PXRD) and FT-IR spectroscopy. PXRD diffractograms were collected using a Bruker DX8 Discover diffractometer (Billerica, MA, USA) with CuKα radiation (λ = 1.54 Å); over the 2 theta range of 20–70° with a step size of 0.024°, a step time of four seconds, and a locked, coupled (theta-theta) geometry. The lattice parameters of the unit cells were determined using Rietveld refinement. A Perkin Elmer Spectrum 1000 spectrometer, Waltham, MA, USA, was used to perform FT-IR experiments. The spectra were recorded using the transmission technique of the KBr tablet with a spectral range of 4000 to 400 cm^−1^ and a spectral resolution of 2 cm^−1^.

To determine the experimental amounts of selenium (IV) and zinc introduced into the structure of HA and DCPD, respectively, ICP-OES was chosen (ICP-OES iCAP 7400 Duo Spectrometer, Thermo Scientific, Waltham, MA, USA). The samples were weighed out, dissolved in Suprapure 63% HNO_3_ (Sigma Aldrich, St. Louis, MO, USA) and diluted adequately with deionised water. The measurements were conducted according to the five-point standard curve and repeated thrice. 

The in vitro cell viability assessment was performed using a normal human foetal osteoblast cell line (hFOB 1.19, ATCC CRL-11372) and a human osteosarcoma cell line (Saos-2, ATCC HTB-85). The hFOB 1.19 cells were cultivated in a 1:1 mixture of Dulbecco’s Modified Eagle Medium/Ham’s F12 culture medium without phenol red (Sigma Aldrich Chemicals) containing 100 U/mL penicillin, 0.1 mg/mL streptomycin, 0.3 mg/mL G418 (Sigma-Aldrich Chemicals, St. Louis, MO, USA), 10% foetal bovine serum (FBS, Pan-Biotech GmbH), and maintained at 34 °C in a humidified atmosphere of 5% CO_2_. The Saos-2 cells were cultivated in McCoy’s 5A medium containing 100 U/mL penicillin, 0.1 mg/mL streptomycin, 15% FBS, and maintained at 37 °C in a humidified atmosphere of 5% CO_2_. 

The cytotoxicity assessment was carried out according to ISO 10993-5:2009 standard (ISO 10993-5, 2009) [30] using 24-h extracts of the samples as previously described [29]. In brief, the hFOB 1.19 and Saos-2 cells were seeded into a 96-well polystyrene plate in 100 µL of a growth medium at a density of 2 × 10^5^ cells/mL and 3 × 10^5^ cells/mL, respectively. After 24 h of cell culture, the growth medium was replaced with 100 µL of previously prepared sample extract. Extract from polystyrene served as a negative control of cytotoxicity (marked as PS control). After 24 h of exposure to the extracts, cell viability was assessed using the MTT test (Sigma-Aldrich Chemicals, St. Louis, MO, USA) as previously described [31]. The results were presented as the percentage of the OD value obtained with the negative control using the following formula: cell viability = (sample OD/negative control OD) × 100%. Next, the results from the MTT assay (*n* = 3) were analysed using GraphPad Prism 8.0.0 Software, San Diego, CA, US. A one-way ANOVA followed by Tukey’s test was applied to determine statistically significant differences among all groups (*p* < 0.05).

### 2.3. Preparation of Porous Granules

**Figure 1:**
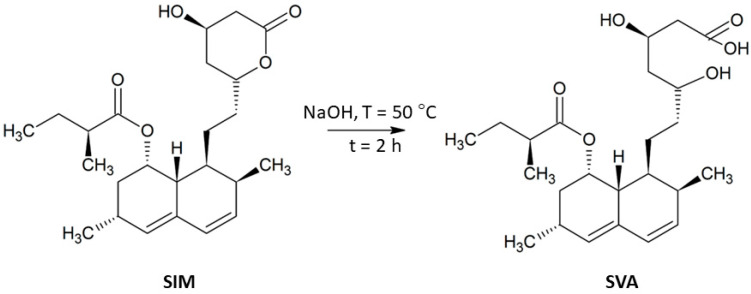
The route of the simvastatin hydrolysis reaction.

In order to prepare porous granules, Zn-DCPD and Se-HA were mixed in equal amounts and homogenised thoroughly in a mortar for 30 min. Subsequently, approximately 2.0 g SA, 2.0 g GEL (both from Sigma Aldrich, St. Louis, MO, USA) (if necessary) and approximately 100 mg of simvastatin (TCI, Tokyo Chemical Industry Co., Tokyo, Japan) were weighed out. At the end of this stage, 1.5% aqueous solution of zinc nitrate hexahydrate and 0.5% ethanolic solution of glutaraldehyde (GAH, Sigma Aldrich, St. Louis, MO, USA) (if necessary) were prepared. The first solution was used as a cross-linking agent in case of the granules containing both SA and GEL as binding materials, whereas the second solution was dedicated to cross-linking of the granules containing only GEL. As mentioned earlier, simvastatin was applied as a model drug in two forms: non-hydrolysed (SIM) and hydrolysed (SVA). The route of the hydrolysis reaction is described elsewhere [14,18] and presented in Figure 1. Briefly, SIM was dissolved in 95% ethanol and treated with 0.1 M NaOH. Then, the solution was heated at 50 °C during 2 h and next, neutralised using 0.1 M HCl to a pH of approximately 7.2.

Once all the reagents were prepared, natural polymers (SA, GEL, or both) were suspended in 50 mL of distilled water at 50 °C. After dissolving the polymers, 4.0 g of mixture of CaPs was added to the slurry and homogenised using a mechanic stirrer. Next, simvastatin was added to the suspension. The prepared viscous slurry was subsequently extruded into the cross-linking solution, which led to immediate gelling and the production of granules. In the case of the beads containing GEL, additional cross-linking with 0.5% GAH solution was needed and was conducted by subsequently soaking the granules in the solution for two hours. Once the extrusion was complete, the remaining slurry was weighed out in order to calculate the amount of simvastatin that had been introduced into the beads. The obtained granules were washed several times with distilled water or ethanol, if necessary, and dried in the open air. The composition of six different types of obtained granules is presented in Table 1.

### 2.4. Characterization of the Granules

To determine the morphology of the granules, SEM microscopy (JSM-6390LV JEOL microscope, JEOL LTD, Tokyo, Japan) was chosen. Both the exterior and interior layers of the beads were evaluated. Prior to the measurements, the samples had been covered with an Au layer.

The evaluation of the specific surface area and the porous structure was conducted using two different techniques: sorption of N_2_, and mercury porosimetry. The first technique was applied to assess the specific surface area, the volume of pores, and their distribution. The measurements were carried out with the analyser of sorption (Micromeritics ASAP 2050). The second technique was used to provide pore diameter distribution, pore volume, and pore surface area. It consisted of the measurement of the volume of mercury intruded into the pore structure under vacuum conditions. The mercury intrusion was evaluated by using a Micromeritics Autopore IV 9510 instrument (Micromeritics, Norcross, GA, USA).

The evaluation of the release kinetics of SIM and SVA was conducted as follows. Firstly, appropriate portions of each type of granule containing SIM or SVA were weighed out and put into 50 mL Falcon tubes. Subsequently, 50 mL of phosphate buffer (pH = 7.4) was poured into each tube. Each tube contained a specific type of granule, and was incubated for a specific period of time. No medium exchange was applied. The samples were incubated at 37 °C with constant stirring at 70 rpm. Once the defined time had elapsed, samples of medium were taken. Due to the fact that simvastatin is poorly soluble in water solutions, the samples were diluted twice, using 95 *v*/*v* % ethanol (Sigma Aldrich, St. Louis, MO, USA). The SIM or the SVA concentration was measured with an ultraviolet visible spectrometer (Shimadzu UV-1800 spectrometer, Shimadzu, Kyoto, Japan) at 238 nm. A 5-point standard curve was applied and the measurements were performed in triplicate.

## 3. Results and Discussion

### 3.1. Characterization of the Obtained CaPs

**Figure 2:**
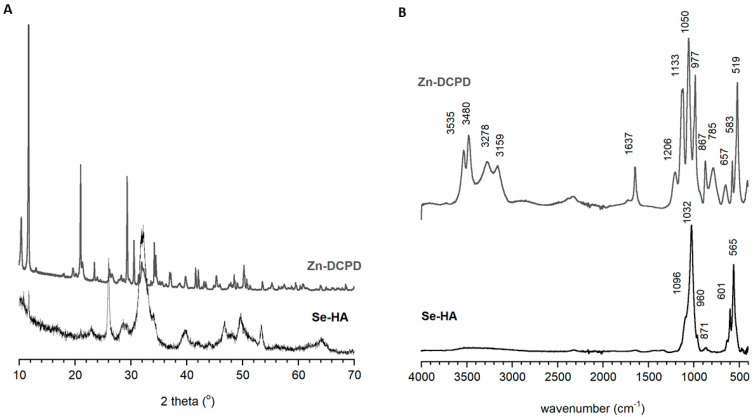
PXRD diffractograms (**A**) and FT-IR spectra (**B**) of the obtained Zn-DCPD and Se-HA samples.

Figure 2A,B show sthe results of the Se-HA and Zn-DCPD powders obtained by PXRD and FT-IR, respectively.

The Se-HA sample powder diffraction pattern is in good agreement with diffraction pattern of hydroxyapatite (JCPD reference card 9-432). However, the reflections are very broad and weakly resolved, typical for nanocrystalline apatite structure [32,33]. The FT-IR spectra of the Se-HA sample contain the characteristic bands for hydroxyapatite: ν_1_ and ν_3_ phosphate bands in the range 1200 to 900 cm^−1^ and the typical ν_4_ two bands at 600 and 575 cm^−1^ [34]. The FT-IR and PXRD results indicate the successful synthesis of homogeneous nanocrystalline hydroxyapatite. The content of selenium in this sample, calculated on the basis of ICP-OES data, was 0.09 *w*/*w* %, which indicates a 64.5% yield.

Similarly, the Zn-DCPD powder is homogeneous in phase; the PXRD pattern is completely consistent with the standard [35]. The FT-IR spectrum is typical for brushite and contains the water bands in the range 3535 to 3159 cm^−1^, and at approximately 1637 cm^−1^, and the phosphate characteristic vibrations bands for HPO_4_^2−^ (1206 cm^−1^ and 785 cm^−1^) [35]. The measured zinc content in this material was 9.15% which was 78.8% of the nominal value.

### 3.2. Biological Properties of CaPs In Vitro

**Figure 3:**
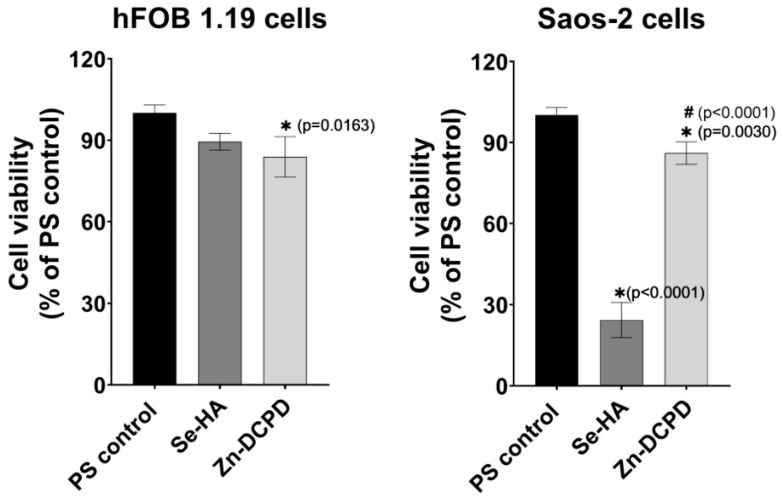
Cytotoxicity assessment by MTT assay after 24-h exposure of foetal osteoblast cell line (hFOB 1.19) cells and human osteosarcoma cell line (Saos-2) cells to extracts of Se-HA powder and Zn-DCPD powder (PS control–negative control of cytotoxicity; * indicates statistically significant results compared to PS control, ^#^ indicates statistically significant results compared to Se-HA; One-way ANOVA followed by Tukey’s test).

Cell viability after exposure to 24-h extracts of fabricated Se-HA and Zn-DCPD powders was evaluated by MTT assay (see Figure 3). The obtained samples were subjected to cytotoxicity assessment using foetal osteoblast cell line (hFOB 1.19) cells and, additionally, to anti-tumour activity assessment using human osteosarcoma cell line (Saos-2) cells. It is worth noting that according to [30], a reduction in cell viability below 70% caused by exposure to 100% extracts of the samples should be considered as a cytotoxic effect [36,37]. As shown in Figure 3, an extract of HA powder modified with Se ions was non-toxic to human osteoblasts (cell viability approx. 89%) and simultaneously exhibited high toxicity to tumour cells (cell viability approx. 24%) as compared to the cells maintained in polystyrene extract (PS control). This is in agreement with previous studies conducted by Bao et al. [38] and Zhou et al. [39] that showed Se-induced cytotoxicity to cancer cells. Moreover, previous studies on selenium-containing hydroxyapatite and its anti-tumour properties clearly show that the key is the appropriate amount of selenium in the materials: unfortunately, too high concentration of selenium is toxic to healthy cells [40]. Therefore, the concentration of Se should be selected in such a way as to ensure effective activity against osteosarcoma cells while being non-toxic to osteoblasts. Thus, it seems that the Se-HA sample meets this requirement.

In turn, as shown in Figure 3, the extract of DCPD powder modified with Zn ions was non-toxic against both human osteoblasts and osteosarcoma cells (cell viability approx. 85%). Thus, in this study, we showed that obtained Se-HA and Zn-DCPD powders are non-toxic against normal human osteoblasts and thereby may be potentially used in bone tissue engineering and regenerative medicine applications, whereas, in the case of Se-HA powder, it may be applied in the therapy of bone cancers. Nevertheless, further in vitro studies need to be conducted to confirm this assumption.

### 3.3. Morphology of the Granules

**Figure 4:**
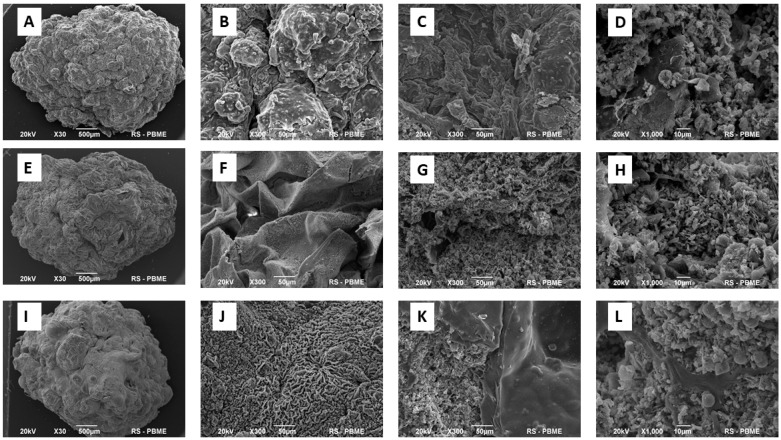
Representative SEM microphotographs of the granules (SIM A–(**A**–**D**); SIM AG–(**E**,**F**); SIM AGG–(**I**–**L**)). Shape of granules (**A**,**E**,**I**), the outer (**B**,**F**,**J**) and the inner (**C**,**D**,**G**,**H**,**K**,**L**) surfaces of the granules.

Figure 4 shows representative SEM microphotographs of the inner and outer surfaces of three types of composite granules after drying (the SIM A, SIM AG and SIM AGG samples). The granules demonstrate somewhat spherical shapes with a diameter of about 3–5 mm and rough morphology. The exterior of the granules exhibits a great roughness with large wrinkles, typical of beads prepared with sodium alginates [32,41]. The addition of GEL into the SA slurry resulted in a slight smoothing of the outer surface and, in contrast, a wrinkling during the drying process (see Figure 4F). The internal cross-section of the granules containing GEL possessed the most homogenous morphology and, at the same time, the largest pores between the grains. Numerous pores of various sizes were visible. According to the results of the analysis, it can be assumed that granules of porous structure were successfully fabricated using a droplet extrusion technique.

### 3.4. Porosity of the Granules

To analyse precisely the porosity of the obtained granules, the next tests were performed with the use of mercury porosimetry.

The total porosity and the pores’ surface area of the materials can be defined as moderate (see Table 2). The structure of each kind of bead: (SIM/SVA) A, (SIM/SVA) AG and (SIM/SVA) AGG) is quite complex, as they contain all types of pores and intergrain spaces, which normally originate during the formulation process. Based on the average diameter of pores (see Table 2), all the beads can be classified as mesoporous materials.

Regarding further analysis of the data presented in Table 2, several facts should be highlighted. The (SIM/SVA) A beads possess a specific surface area approximately twice that of the other bead types. In turn, (SIM/SVA) AGG) beads present the lowest surface area of pores. (SIM/SVA) AG beads, however, stand out from the other granules. They simultaneously exhibit both the highest total percentage of mesopores and the lowest average diameter of the pores. This suggests that their porous structure is the most complex. This complexity is based on simultaneously possessing a significant number of mesopores and micropores, which contributes to the general decrease of the average diameter of pores and their total volume.

### 3.5. SIM and SVA Release Profiles

**Figure 5:**
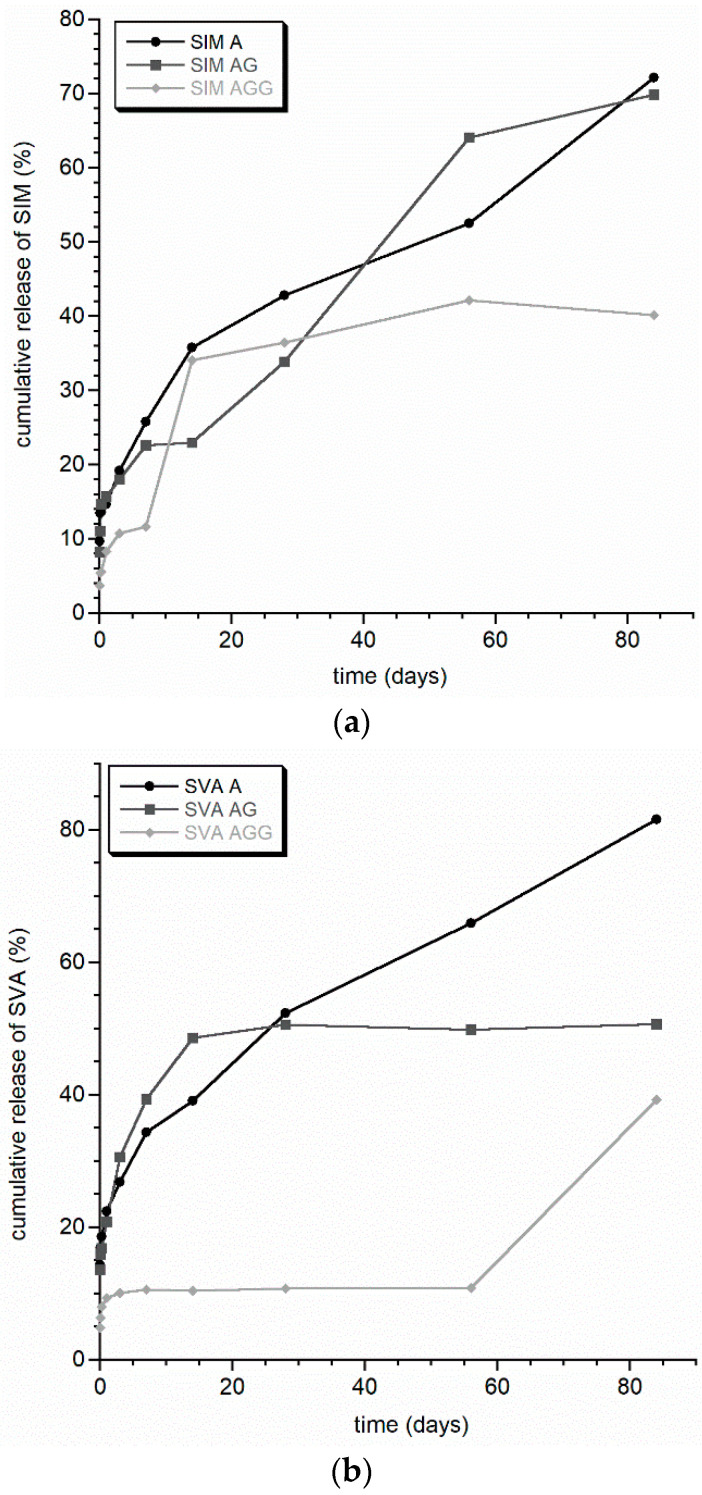
SIM (**a**) and SVA (**b**) release curves from A, AG, and AGG granules.

The results of the kinetics of release of simvastatin (SIM and SVA) from the obtained granules are presented in Figure 5. Although all six profiles are slightly different, they all show a sustained release of simvastatin for about three months (see Figure 5). No burst release was observed, which may be ascribed to the homogenous dispersion of drug molecules within the beads. It is likely that the mechanism of the release of simvastatin from the fabricated matrices is modulated by a swelling-dissolution-erosion process. Thus, the more permeable the beads, the faster the release. The highest cumulative release of the model drug was observed in the (SIM/SVA) A granules and was around 80 *w/w* % of the initial loading. This may be attributed to their significant surface area (see Table 2), which increases their permeability and facilitates the release process of simvastatin. This is consistent with literature reports, which consider alginate beads to be highly permeable [12,42].

The overall cumulative release did not vary significantly between the two different forms of simvastatin, lipophilic (SIM) or hydrophilic (SVA). Nevertheless, it should be concluded that the cross-linking between covalent gelatin and glutaraldehyde significantly delays the release of the model drug. This may be related to the low pore’s surface of the (SIM/SVA) AGG beads, which makes them the least degradable. Three profiles (SIM AG, SIM AGG and SVA AGG) exhibited more than one plateau. The authors believe that in case of the (SIM/SVA) AG beads, this may be attributed to their bimodal distribution of pores, as already mentioned. In turn, due to the fact that both SIM AGG and SVA AGG possess the same pore structure, the existence of two plateaux can be related to the electrostatic interactions between both forms of simvastatin and covalently crosslinked gelatin. Interestingly, only in the case of the (SIM/SVA) AGG beads was a slight difference between the release profile of SIM and SVA observed. Although the overall release of the model drug was at a similar level, for SVA the first plateau was observed for a significantly longer period of time. This suggests possible stronger electrostatic bonds between SVA and crosslinked GEL.

## 4. Conclusions

In this work, 3D porous composite granules consisting of bioceramic matrix and natural polymers were successfully fabricated using a droplet-extrusion technique. The composition of the granules varied slightly as they consisted of two different CaPs materials (Se-HA and Zn-DCPD) and also two different natural polymers, sodium alginate (SA) and gelatin (GEL).

The type of polymer used had a great impact on the porosity of the granules, which in turn influenced the release kinetics of simvastatin, which had been introduced into the granules to evaluate their potential as drug carriers targeting bones. The results of release kinetics tests turned out to be promising, as the release of the model drug was sustained and no burst release was observed. Prior to the fabrication of the granules, cytotoxicity assessment of the output powders was conducted. The results of the assay proved to be of extreme importance as they revealed a Se-HA material, which was non-toxic to human osteoblasts (hFOB 1.19) and simultaneously exhibited high toxicity to tumour cells (Saos-2).

In summary, this preliminary study is a starting point to design and fabricate CaPs-based drug delivery systems targeting bones with additional bone healing and anti-tumour potential.

## Figures and Tables

**Table 1:** The composition of the different types of obtained granules.

Components Granules	Se-HA (*w*/*v* %)	Zn-DCPD (*w*/*v* %)	SA (*w*/*v* %)	GEL (*w*/*v* %)	Cross−Linking Agent	
					1.5% Zn^2+^ (aq.)	0.5% GAH (et.)
SIM A	4.0	4.0	4.0	−	+	−
SIM AG	4.0	4.0	2.0	2.0	+	−
SIM AGG	4.0	4.0	2.0	2.0	+	+
SVA A	4.0	4.0	4.0	−	+	−
SVA AG	4.0	4.0	2.0	2.0	+	−
SVA AGG	4.0	4.0	2.0	2.0	+	+

**Table 2:** Porosity results of the obtained granules measured by the Hg intrusion technique and N_2_ sorption.

Granules	Specific Surface [m^2^/g]	Pores’ Surface [m^2^/g]	Total X56-p[s0poporosity [%]	Total Pores’ Volume [cm^3^/g]	Mesopores’ Percentage [%]	Average Pores’ Diameter [nm]
SIM/SVA A	13.8	51	43	0.46	37	36
SIM/SVA AG	6.2	51	39	0.34	43	27
SIM/SVA AGG	7.0	44	43	0.42	33	38

## Data Availability

Not applicable.

## References

[1] Habraken W., Habibovic P., Epple M., Bohner M. (2016). Calcium phosphates in biomedical applications: Materials for the future?. Mater. Today.

[2] Lu Y., Yu H., Chen C. (2018). Biological properties of calcium phosphate biomaterials for bone repair: A review. RSC Adv..

[3] Dorozhkin S.V., Epple M. (2002). Biological and Medical Significance of Calcium Phosphates. Angew. Chem. Int. Ed..

[4] Vallet-Regi M., Gonzalez-Calbet J.M. (2004). Calcium phosphates as substitution of bone tissue. Prog. Solid State Chem..

[5] Chahkandi M., Mirzaei M. (2017). Structural and particle size evolution of sol-gel derived nanocrystalline hydroxyapatite. J. Iran. Chem. Soc..

[6] Kolmas J., Krukowski S., Laskus A., Jurkitewicz M. (2016). Synthetic hydroxyapatite in pharmaceutical applications. Ceram. Int..

[7] Ginebra M.-P., Canal C., Espanol M. (2012). Calcium phosphate cements as drug delivery materials. Adv. Drug Deliv. Rev..

[8] Supova M. (2015). Substituted hydroxyapatites for biomedical applications: A review. Ceram. Int..

[9] Laskus A., Kolmas J. (2017). Ionic substitution in non-apatitic calcium phosphates. Int. J. Mol. Sci..

[10] Bernhardt A., Despang F., Lode A., Demmler A., Hänke T., Gelinsky M. (2009). Proliferation and osteogenic differentiation of human bone marrow stromal cells on alginate–gelatine–hydroxyapatite scaffolds with anisotropic pore structure. J. Tissue Eng. Regen. Med..

[11] Li H., Jiang F., Ye S., Wu Y., Zhu K., Wang D. (2016). Bioactive apatite incorporated alginate microspheres with sustained drug-delivery for bone regeneration application. Mat. Sci. Eng. C.

[12] Ferreira Almeida P., Almeida A.J. (2004). Cross-linked alginate-gelatin beads: A new matrix for controlled release of pindolol. J. Control. Release.

[13] Levingstone T.J., Herbaj S., Dunne N.J. (2019). Calcium phosphate nanoparticles for therapeutic applications in bone regeneration. Nanomaterials.

[14] Canal C., Khurana K., Gallinetti S., Bhatt S., Pulpytel J., Arefi-Khonsari F., Ginebra M.-P. (2016). Design of calcium phosphate scaffolds with controlled simvastatin release by plasma polymerisation. Polymer.

[15] Mestres G., Kugiejko K., Pastorino D., Unosson J., Öhman C., Ott M.K., Ginebra M.-P., Persson C. (2016). Changes in the drug release pattern of fresh and set simvastatin-loaded brushite cement. Mat. Sci. Eng. C.

[16] Kheirallah M., Almeshally H. (2016). Simvastatin, dosage and delivery system for supporting bone regeneration, an update review. J. Oral Maxillofac. Surg. Med. Pathol..

[17] Oryan A., Kamali A., Moshiri A. (2015). Potential mechanisms and applications of statins on osteogenesis: Current modalities, conflicts and future directions. J. Control. Release.

[18] Kaesemeyer W.H., Caldwell R.B., Huang J., Caldwell R.W. (1999). Pravastatin Sodium Activates Endothelial Nitric Oxide Synthase Independent of Its Cholesterol-Lowering Actions. JACC.

[19] Lau C.P.Y., Fung C.S.H., Wong K.C., Wang Y., Huang L., Tsui S.K.W., Lee O.K., Kumta S.M. (2020). Simvastatin possesses antitumor and differentiation-promoting properties that affect stromal cells in giant cell tumor of bone. J. Orthop. Res..

[20] Kany S., Woschek M., Kneip N., Sturm R., Kalbitz M., Hanschen M., Relja B. (2018). Simvastatin exerts anticancer effects in osteosarcoma cell lines via geranylgeranylation and c-Jun activation. Int. J. Oncol..

[21] Di Bello E., Zwergel C., Mai A., Valente S. (2020). The innovative potential of statins in cancer: New targets for new therapies. Front. Chem..

[22] Mandal C.C., Ghosh-Choudhury N., Yoneda T., Choudhury G.G., Ghosh-Choudhury N. (2011). Simvastatin prevents skeletal metastasis of breast cancer by an antagonistic interplay between p53 and CD44. J. Biol. Chem..

[23] Cuozzo R.C., Miguez da Rocha Leao M.H. (2014). Zinc alginate-hydroxyapatite composite microspheres for bone repair. Ceram. Int..

[24] Shepherd D., Mucalo M. (2015). Zinc-substituted hydroxyapatite for the inhibition of osteoporosis. Hydroxyapatite (Hap) for Biomedical Applications.

[25] Uskokovic V., Iyer M.A., Vu V.M. (2017). One ion to rule them all: Combined antibacterial, osteoinductive and anticancer properties of selenite-incorporated hydroxyapatite. J. Mater. Chem. B.

[26] Laskus A., Zgadzaj A., Kolmas J. (2021). Synthesis and physicochemical characterization of Zn-doped brushite. Ceram. Int..

[27] Laskus A., Zgadzaj A., Kolmas J. (2019). Zn^2+^ and SeO_3_^2−^ co-substituted hydroxyapatite: Physicochemical properties and biological usefulness. Ceram. Int..

[28] Kolmas J., Oledzka E., Sobczak M., Nałecz-Jawecki G. (2014). Nanocrystalline hydroxyapatite doped with selenium oxyanions: A new material for potential biomedical applications. Mater. Sci. Eng. C.

[29] Kolmas J., Pajor K., Pajchel L., Przekora A., Ginalska G., Oledzka E., Sobczak M. (2017). Fabrication and physicochemical characterization of porous composite microgranules with selenium oxyanions and risedronate sodium for potential applications in bone tumours. Int. J. Nanomed..

[30] (2009). ISO 10993-5 Biological Evaluation of Medical Devices—Part 5: Tests for In Vitro Cytotoxicity.

[31] Przekora A., Czechowska J., Pijocha D., Ślósarczyk A., Ginalska G. (2014). Do novel cement-type biomaterials reveal ion reactivity that affects cell viability in vitro?. Centr. Eur. J. Biol..

[32] Bouropoulos N., Stampolakis A., Mouzakis D.E. (2010). Dynamic mechanical properties of calcium-alginate- hydroxyapatite nanocomposite hydrogels. Sci. Adv. Mater..

[33] Pajchel Ł., Kowalska V., Smoleń D., Kedzierska A., Pietrzykowska E., Lojkowski W., Kolodziejski W. (2013). Comprehensive structural studies of ultra-fine nanocrystalline calcium hydroxyapatite using MAS NMR and FT-IR spectroscopic methods. Mater. Res. Bull..

[34] Boanini E., Silingardi F., Gazzano M., Bigi A. (2021). Synthesis and hydrolysis of brushite (DCPD): The role of ionic substitution. Cryst. Growth Des..

[35] Kumar M., Xie J., Chittur K., Riley C. (1999). Transformation of modified brushite to hydroxyapatite in aqueous solution: Effects of potassium solution. Biomaterials.

[36] Gustavsson J., Ginebra M.P., Engel E., Planell J. (2011). Ion reactivity of calcium-deficient hydroxyapatite in standard cell culture media. Acta Biomater..

[37] Malafaya P.B., Reis R.L. (2009). Bilayered chitosan-based scaffolds for osteochondral tissue engineering: Influence of hydroxyapatite on in vitro cytotoxicity and dynamic bioactivity studies in a specific double-chamber bioreactor. Acta Biomater..

[38] Bao P., Chen Z., Tai R.-Z., Shen H.-M., Martin F.L., Zhu Y.-G. (2015). Selenite-induced toxicity in cancer cells is mediated by metabolic generation of endogenous selenium nanoparticles. J. Proteome Res..

[39] Zhou Z.-F., Sun T.-W., Qin Y.-H., Zhu Y.J., Jiang Y.Y., Zhang Y., Liu J., Wu J., He S., Chen F. (2019). Selenium-doped hydroxyapatite biopapers with an anti-bone tumor effect by inducing apoptosis. Biomater. Sci..

[40] Barbanente A., Palazzo B., Esposti L.D., Adamiano A., Iafisco M., Ditaranto N., Migoni D., Gervaso F., Nadar R., Ivanchenko P. (2021). Selenium-doped hydroxyapatite nanoparticles for potential application in bone tumour therapy. J. Inorg. Biochem..

[41] Zhang J., Wang Q., Wang A. (2010). In situ generation of sodium alginate/hydroxyapatite nanocomposite beads as drug-controlled release matrices. Acta Biomater..

[42] Venkatesan J., Bhatnagar I., Manivasagan P., Kang K.H., Kim S.K. (2015). Alginate composites for bone tissue engineering: A review. Int. J. Biol. Macrobiol..

